# Viability determination data for odontoblast-like cells exposed to resin monomers

**DOI:** 10.1016/j.dib.2020.106684

**Published:** 2020-12-23

**Authors:** Paula Alejandra Baldion, Myriam L. Velandia-Romero, Jaime E. Castellanos

**Affiliations:** a Grupo de Investigaciones Básicas y Aplicadas en Odontología, Departamento de Salud Oral, Facultad de Odontología, Universidad Nacional de Colombia, Bogotá, Colombia; b Grupo de Virología, Universidad El Bosque, Bogotá, Colombia

**Keywords:** Hydroxyethyl methacrylate, Triethylene glycol dimethacrylate, Odontoblasts, Cytotoxicity, Cell viability, Calcein, MTT, Resazurin

## Abstract

Data in this article are associated with our research article “Dental Resin Monomers Induce Early and Potent Oxidative Damage on Human Odontoblast-like Cells.” Dental adhesives are polymeric compounds consisting of several chemical substances, including resin monomers, such as 2-hydroxyethyl methacrylate (HEMA) and triethylene glycol dimethacrylate (TEGDMA), together with other comonomers, making up the organic matrix of the adhesive and whose composition is based on the methyl methacrylate chemistry. The release of residual monomers, susceptible to biodegradation, acts as a source of bioactive compounds, which can interact with tissues and induce a cytotoxic cellular response. The most used techniques to evaluate cytotoxicity, proliferation, or metabolic activity of cells exposed to different substances, are MTT and resazurin. Each chemistry evaluates cell viability differently, so the data obtained could vary depending on the technique sensitivity to detect changes in cell metabolism. The objective of this article was to present viability data as a function of the metabolic activity in human odontoblast-like cells (hOLCs), exposed to 3, 6, 9, and 12 mM HEMA, or 0.75, 1.5, 3, and 6 mM TEGDMA evaluated by the MTT, and resazurin techniques in the first 24 hours of exposure, at different time points. The absorbance data for the MTT test and the fluorescence intensity for the resazurin test were obtained by spectrometry. SIMSTAT software 2.6.5 for Windows was used to confirm the normal data distribution (Levene's test). Subsequently, an analysis of variance (one-way ANOVA) was performed to compare the control with each HEMA and TEGDMA concentration. Where a *p* < 0.05 indicated a high F value, a Fisher's least significant differences post-hoc analysis was performed, using an alpha value < 0.05. Data from the different time points were compared with a Student's t-test for each concentration. These data may be useful to compare the cytotoxic response of hOLCs with other cell types or the cell response to other resin monomers.

## Specifications Table

**Table:** 

Subject	Dentistry, Oral Surgery and Medicine
Specific subject area	Cytotoxicity of dental resin monomers
Type of data	Table Figure
How data were acquired	Absorbance and fluorescence intensity was detected using a spectrometer (Infinite M200, Tecan; Männedorf, Switzerland)
Data format	Raw Analyzed
Parameters for data collection	The assays were performed in an Odontoblast-like Cells (OLCs) model obtained through the differentiation of human dental pulp stem cells (hDPSC) [1]. Cells (25 × 10^3^) were seeded in 96-well plates and exposed to 3, 6, 9, and 12 mM HEMA, or 0.75, 1.5, 3, and 6 mM TEGDMA. Cell viability data are presented after a 4, 8, 12, and 24 h exposure for the MTT assay; 30 and 90 min, 3, 6, 9, 12, 18, and 24 h for the resazurin assay.
Description of data collection	Microsoft Office Excel 2010 (Microsoft Corporation, Redmond, Washington, USA) was used for the databases construction. The data are shown as three time points of cell response to monomers: an early (30 and 90 min), medium (4 and 8 h, for MTT, or 3, 6, and 9 h, for resazurin), and late behaviour (12, 24 and 48 h, for MTT, or 12, 18, and 24 h, for resazurin). SIMSTAT software 2.6.5 for Windows was used to confirm the normal data distribution (Levene's test). Subsequently, an analysis of variance (one-way ANOVA) was performed to compare the control with each HEMA and TEGDMA concentration. Where *p* < 0.05 indicated a high *F* value, a Fisher's Least Significant Difference post-hoc analysis was performed, using an alpha value < 0.05. Data from the different time points were compared with a Student's t-test for each concentration.
Data source location	Universidad Nacional de Colombia, Bogotá, Colombia.
Data accessibility	The raw data are deposited in Mendeley Data, V1, https://doi.org/10.17632/hbb8f9nbcv.1[2].
Related research article	P.A. Baldion, M.L. Velandia-Romero, J.E. Castellanos. Dental Resin Monomers Induce Early and Potent Oxidative Damage on Human Odontoblast-like Cells. Chem Biol Interact. 333 (2021) 109336 [3].

## Value of the Data

• These data provide information obtained using alternative methods for cell viability assays to determine the cytotoxicity of dental monomers.
• These data may be useful for comparing the cytotoxic response of hOLCs with other cell types and to compare the cell response to other resin monomers or dental adhesive systems.
• An understanding of the toxicity mechanisms resulting from resin monomer exposure will allow for a better estimation of the consequences associated to the clinical use of these monomers, allowing for the development of improved strategies for the restoration of dental structures.
• The data obtained can support research in cell biology and the biocompatibility evaluation of dental materials, to guide research and development of new techniques and materials that guarantee a better performance of dental adhesive restorations.

## 1 Data Description

With respect to the effect of 2-hydroxyethyl methacrylate (HEMA) and triethyleneglycol dimethacrylate (TEGDMA) exposure on metabolic activity and cell survival of the human odontoblast-like cell, data used can be divided into raw and analyzed.

Regarding the methods used for evaluating the cell response to monomers obtained on an in vitro cell model of OLCs differentiated from hDPSC, the raw data were gathered in several worksheets as follows: cell viability, metabolic activity, and membrane integrity evaluated by calcein, MTT (3-(4,5-dimethylthiazol-2-yl)-2,5-diphenyltetrazolium bromide), resazurin (7-Hydroxy-3*H*-phenoxazin-3-one 10-oxide), and lactate dehydrogenase assays; oxidative damage assessed through three methods: intracellular reactive oxygen species using 2′, 7′-dichlorodihydrofluorescein diacetate assay [4], mitochondrial membrane potential (Δψm) evaluation using tetramethylrhodamine ethyl ester assay, and lipid peroxidation detection by quantifying the malondialdehyde (MDA) molecule; activation of the apoptotic death determined by caspase-3 activity; and cell survival assessed through antioxidant enzymes (catalase and Heme-oxygenase 1) expression by quantitative polymerase chain reaction (qPCR). Raw data on the cell response assessed for each assay are shown in tables where the following information is at display: monomer, time points, untreated controls, treated groups (3, 6, 9, and 12 mM HEMA, or 0.75, 1.5, 3, and 6 mM TEGDMA), intraexperiment and inter-experiment replicas, mean, and standard deviation. This whole dataset is available on the online Mendeley Data repository under the title “Data of the effect of dental resin monomers on human odontoblast-like cells” [2].

In turn, analyzed data on the aforementioned methods are displayed in research article [3] and summarized in Table 1, except for MTT and resazurin assays, which are to be found below.

**Table 1:** Data description available at Mendeley Data repository.

Excel worksheet	Method	Time Points	n	Data	Measurement units
Calcein	Cell viability evaluation by calcein	30, 90 min 3, 6, 9, 12, 18, 24 h	9	Measure of cytoplasmatic esterases activity	Fluorescence intensity expressed in relative fluorescence units (RFU)
LDH	Lactate dehydrogenase (LDH) release assay	30, 90 min 3, 6, 9, 12, 18, 24 h	6	LDH released by permeabilization of the plasma membrane	Measured absorbance, which is proportional to the amount of LDH released in the supernatant
ROS	2′,7′-dichlorodihydrofluorescein diacetate technique	30, 90 min 3, 6, 9, 12, 18, 24 h	9	Intracellular accumulation of Reactive Oxygen Species	Fluorescence intensity expressed in RFU
TMRE	Tetramethylrhodamine ethyl ester (TMRE) assay	30, 90 min 3, 6, 9, 12, 18, 24 h	9	Changes in Mitochondrial membrane potential (Δψm)	Fluorescence intensity expressed in RFU
MDA	Lipid peroxidation assay	30, 90 min 3, 6, 9, 12, 18, 24 h	4	Quantification of the MDA (main by-product of the oxidative degradation of lipid membrane)	Results were first expressed in RFU, and then converted to MDA (nmol)
qPCR	Quantitative polymerase chain reaction	3, 9, 18 h	9	Evaluation of antioxidant enzyme expression for Catalase (CAT) and Heme oxigenase-1 (HO-1)	Fold change as the expression of the antioxidant enzyme genes relative to the internal control (β-actin)
Caspase-3	Caspase-3 activity assay	3, 6, 9, 12, 18, 24 h	6	Induction of apoptotic cell death due to caspase-3 activation	Caspase-3 activity expressed in μmol of p Nitroalinides (pNA) × min/mL of cell lysate
MTT	MTT assay	4, 8, 12, 24, 48 h	18	Metabolic activity evaluation	Measured absorbance, which is proportional to the number of viable, metabolically active cells
Resazurin	Resazurin technique	30, 90 min 3, 6, 9, 12, 18, 24 h	9	Metabolic activity evaluation	Fluorescence intensity (expressed in RFU), which is proportional to the number of viable, metabolically active cells

**Table 2:** Comparison of metabolic activity percentage decrease after 24 h exposure to the maximum HEMA and TEGDMA concentrations.

Assay	HEMA*	SD	TEGDMA⁎⁎	SD
MTT	54.9%	17.9%	34.7%	5.8%
Resazurin	43,2%	4.8%	31.6%	2%

SD: Standard deviation

⁎ HEMA a 12 mM

⁎⁎ TEGDMA a 6 mM.

**Table 3:** Comparison of metabolic activity percentage decrease after 24 h exposure to the same HEMA and TEGDMA concentrations.

Assay	HEMA*	SD	TEGDMA⁎⁎	SD
MTT	11.7%	6.5%	34.7%	5.8%
Resazurin	31.8%	2.3%	31.6%	2%

SD: Standard deviation

⁎ HEMA a 6 mM

⁎⁎ TEGDMA a 6 mM.

Analyzed data on MTT and resazurin assays are the result of experiments carried out on isolated cells that had been differentiated using odontogenic differentiation medium (containing TGF-β1) [1], with intraexperiment replications for data validation. Collected data show the effects of resin monomers on cell viability. Cells were treated with different concentrations of HEMA and TEGDMA for 4, 8, 12, 24 and 48 h and were evaluated by the technique of MTT. The MTT assay, although the most frequently used, only allowed us to observe cellular metabolism changes 8 h after exposure. Therefore, the effect of monomers was assessed using the resazurin technique and evaluating the effect of the exposure to monomers at shorter time intervals, namely, 30 and 90 min, and 3, 6, 9, 12, 18, and 24 h. The absorbance values and the fluorescence intensity were obtained by spectrometry at the specific wavelengths for each test.

## 2 Experimental Design, Materials and Methods

### 2.1 Exposure conditions

In the model of hOLCs differentiated from mesenchymal stem cells of dental pulp [1], viability was determined as a function of metabolic activity using the MTT and resazurin, compared to the monomers concentrations and the exposure time. The cells were seeded in 96-well plates and, following their adhesion, were exposed to 3, 6, 9, and 12 mM HEMA (Sigma-Aldrich, St. Louis, MO, USA), or 0.75, 1.5, 3, and 6 mM TEGDMA (Sigma-Aldrich). The evaluation times varied according to the technique. For the MTT assay, cell viability was evaluated after 4, 8, 12, and 24 h. For the resazurin assay, cell viability was evaluated after 30 and 90 min, 3, 6, 9, 12, 18, and 24 h.

### 2.2 MTT assay

**Fig. 1:**
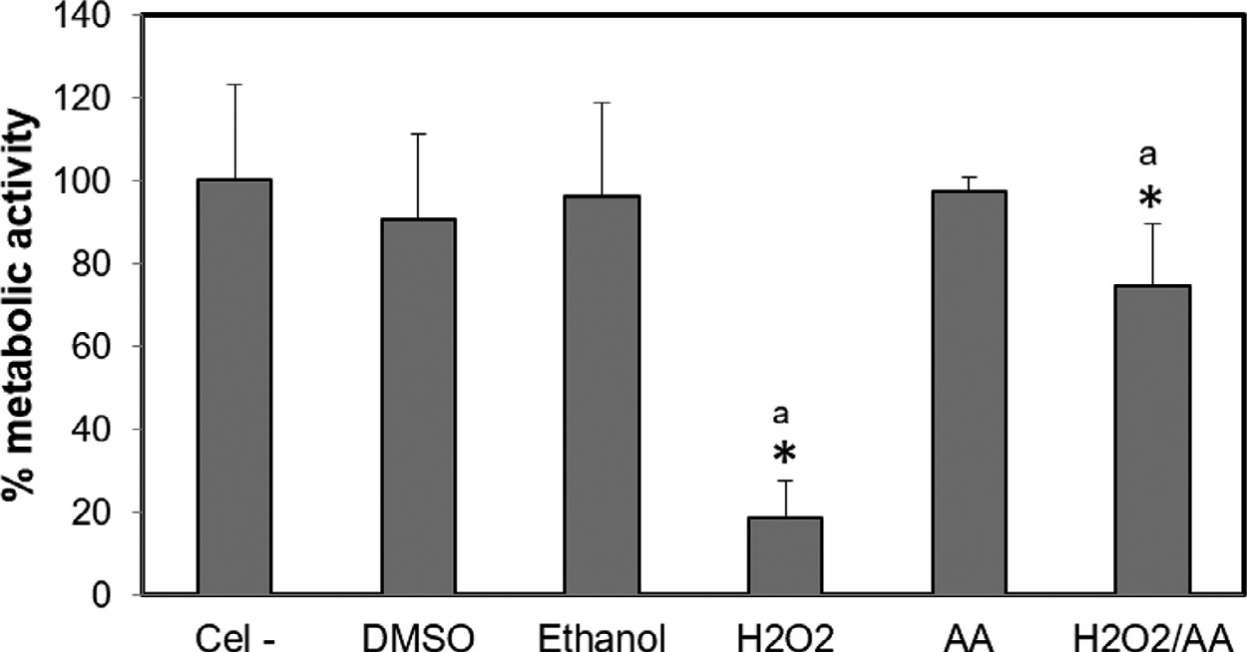
Control groups for the MTT assay. The control groups for the metabolic activity assays were untreated cells (Cel-), cells exposed to the maximum solvent concentrations (0.22% DMSO or ethanol), and cells exposed to 1 mM hydrogen peroxide (H_2_O_2_) diluted in medium without phenol red with 10% SFB for 4 h (positive control). To evaluate the free radicals’ role on cell cytotoxicity, cells were also incubated with ascorbic acid (AA) (50 μg/mL), following a previously reported protocol by Samuelsen et al. (2007) [4], or H_2_O_2_ with AA. Data are presented as the mean ± SD of three independent experiments, each with six replicates (n = 18). Each treatment was compared to the 100% metabolic activity of the negative control (untreated cells). Asterisks indicate significant differences compared to the negative control (*p* < 0.05). The lowercase letter (a) indicates significant differences between treatment groups (*p* < 0.05).

**Fig. 2:**
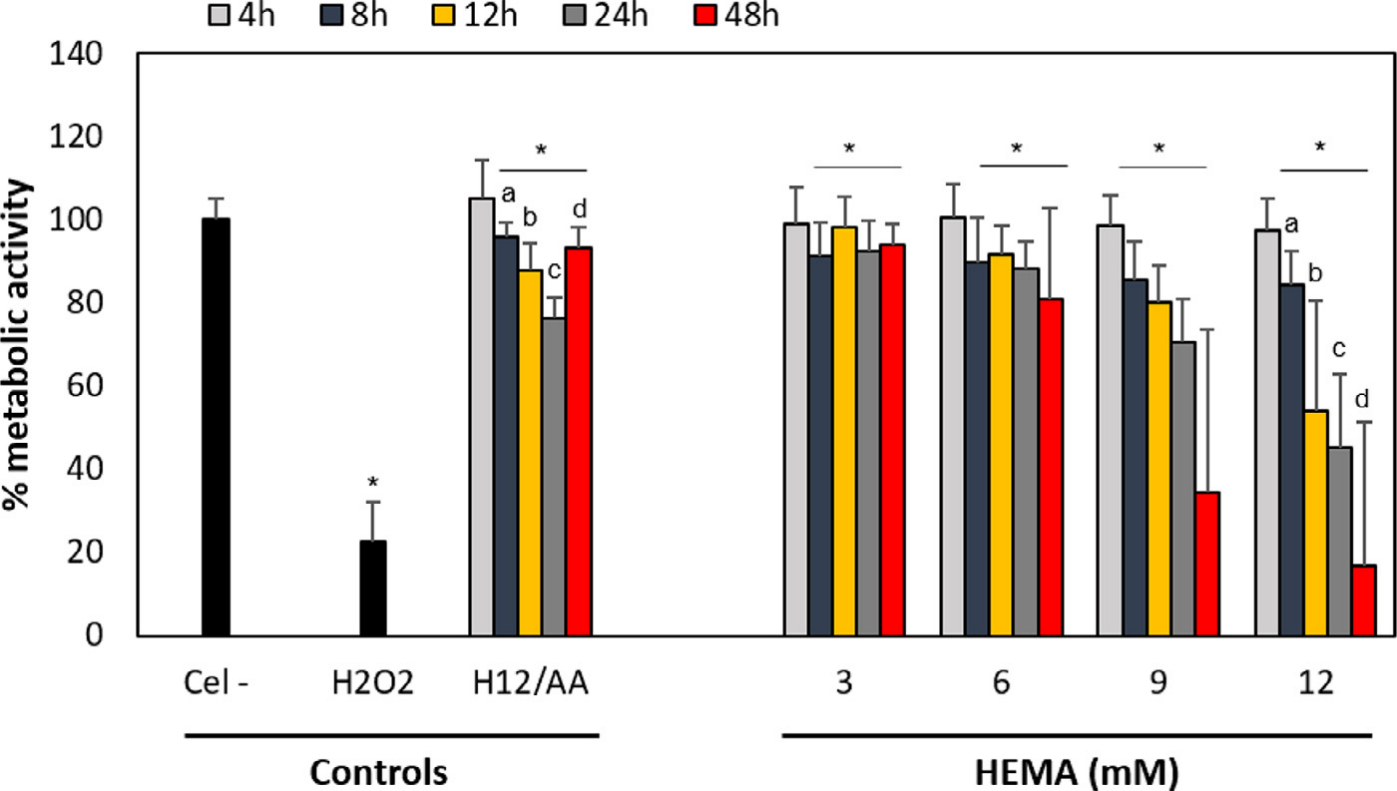
Effect of HEMA on metabolic activity in hOLCs, measured by the MTT assay. Cells exposed to 3, 6, 9, and 12 HEMA for 4, 8 h. (B) Cells exposed to 3, 6, 9, and 12, 12, 24, and 48 h. Mitochondrial metabolic activity was detected after monomers exposure using the MTT assay and quantified by spectrophotometry at 570 nm. The decreased cell capacity to metabolically reduce MTT to formazan was evident after 8 h exposure to 3, 6, 9, and 12 HEMA. Data are shown as the mean ± SD of three independent experiments, each with six replicates (*n* = 18). Each treatment was compared to the % metabolic activity of the negative (Cel-) and positive (1 mM H_2_O_2_ for 4 h) controls, and of the cells exposed to the highest HEMA concentration (12 mM) with 50 μg/mL of ascorbic acid (H12/AA) for each time point. Asterisks indicate significant differences compared to the negative control (*p* < 0.05). Lowercase letters (a - d) indicate significant differences between treatment groups (*p* < 0.05).

**Fig. 3:**
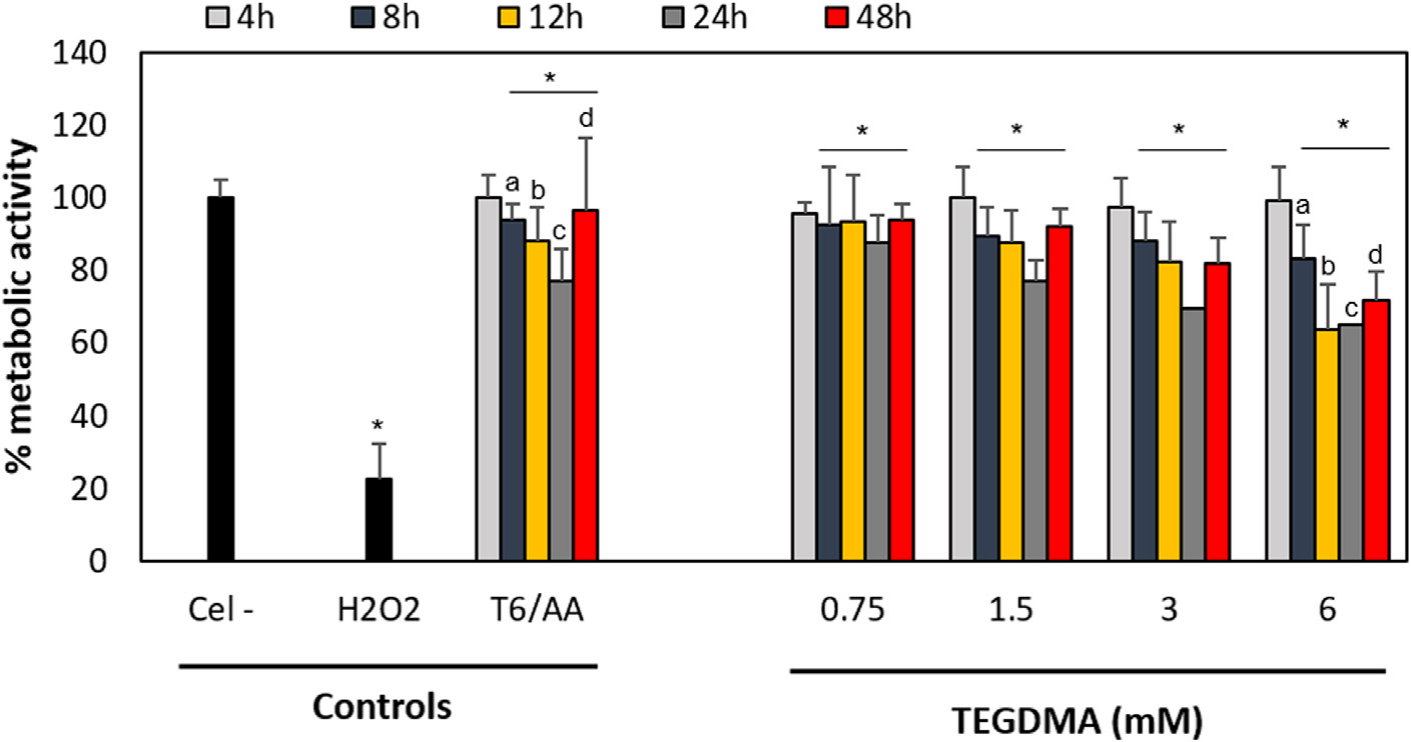
Effect of TEGDMA on metabolic activity in hOLCs, measured by the MTT assay. Cells exposed to 0.75, 1.5, 3, and 6 TEGDMA for 4, 8 h. (B) Cells exposed to 0.75, 1.5, 3, and 6, 12, 24, and 48 h. All TEGDMA concentrations reduced the rate of cellular metabolism from 8 to 48 h. Data are shown as the mean ± SD of three independent experiments, each with six replicates (*n* = 18). Each treatment was compared to the % metabolic activity of the negative (Cel-) and positive (100 µM H_2_O_2_ for 4 h) controls, and of the cells exposed to the highest TEGDMA concentration (6 mM) with 50 μg/mL of ascorbic acid (AA/6) for each time point. Asterisks indicate significant differences compared to the negative control (*p* < 0.05). Lowercase letters (a - d) indicate significant differences between treatment groups (*p* < 0.05).

A colorimetric assay was used to quantify cell viability. The biochemical reaction is based on the mitochondrial enzymes’ activity, which are active in living cells. The hOLCs were seeded at a cell density of 25 × 10^3^ cells/well in a 96-well plate and incubated at 37 °C in a humidified atmosphere containing 5% CO_2_ for 20 h to allow adhesion. Subsequently, the cells were exposed to HEMA and TEGDMA at different concentrations and times. MTT solution (M-5556 Sigma-Aldrich) was added (100 µL) at a final concentration of 0.5 mg/mL in each well, as previously reported by Mossmann [5]. The formazan accumulated in each well after a 3 h incubation at 37 °C in a 5% CO_2_ atmosphere. The MTT solution was then removed without washing and the crystals formed were dissolved in 100 μL DMSO for 15 min. The absorbance was determined using a spectrophotometer (Infinite M200, Tecan; Männedorf, Switzerland) at 570 nm. Untreated cells, cells treated with the maximum concentration of DMSO or ethanol (0.22%), and cells exposed to 1 mM hydrogen peroxide (H_2_O_2_), were taken as controls. The optical density of the untreated cells was taken as 100% viability or metabolic activity. There was a correlation between the absorbance value and cell viability or metabolic activity. Three independent cultures were analyzed with six replicates per condition (*n* = 18) (Figs 1–3).

**Fig. 4:**
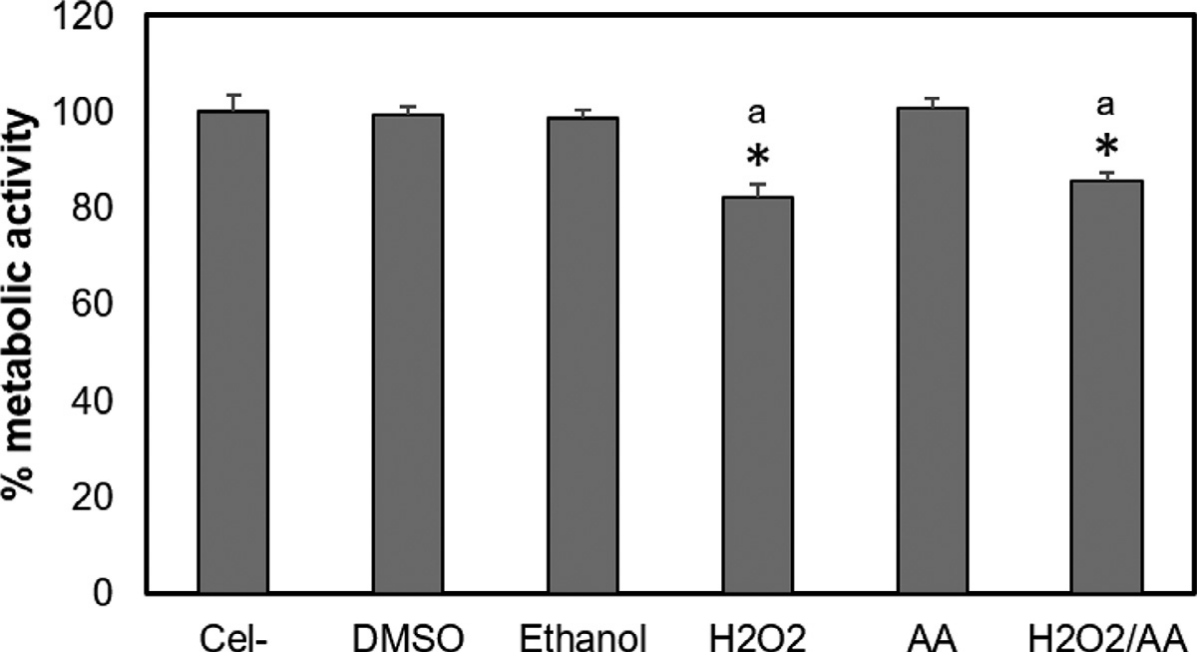
Control groups for the resazurin technique. The control groups for the viability assays were untreated cells (Cel -), cells exposed to the maximum DMSO or ethanol concentrations, and cells exposed to 100 µM hydrogen peroxide (H_2_O_2_) diluted in medium without red phenol with 10% FBS for 4 h (positive control). To evaluate the free radicals’ role on cell cytotoxicity, cells were also incubated with 50 μg/mL ascorbic acid (AA) only or AA together with the highest concentration of each monomer (12 mM HEMA (H12/AA), 6 mM TEGDMA (T6/AA), and H_2_O_2_ with AA). Results are shown as the mean ± SD of three independent experiments, each with three replicates (*n* = 9). Each treatment was compared to the 100% metabolic activity of the negative control (untreated cells). Asterisks indicate significant differences compared to the negative control (*p* < 0.05). The lowercase letter (a) indicates significant differences between treatment groups (*p* < 0.05).

### 2.3 Resazurin assay

**Fig. 5:**
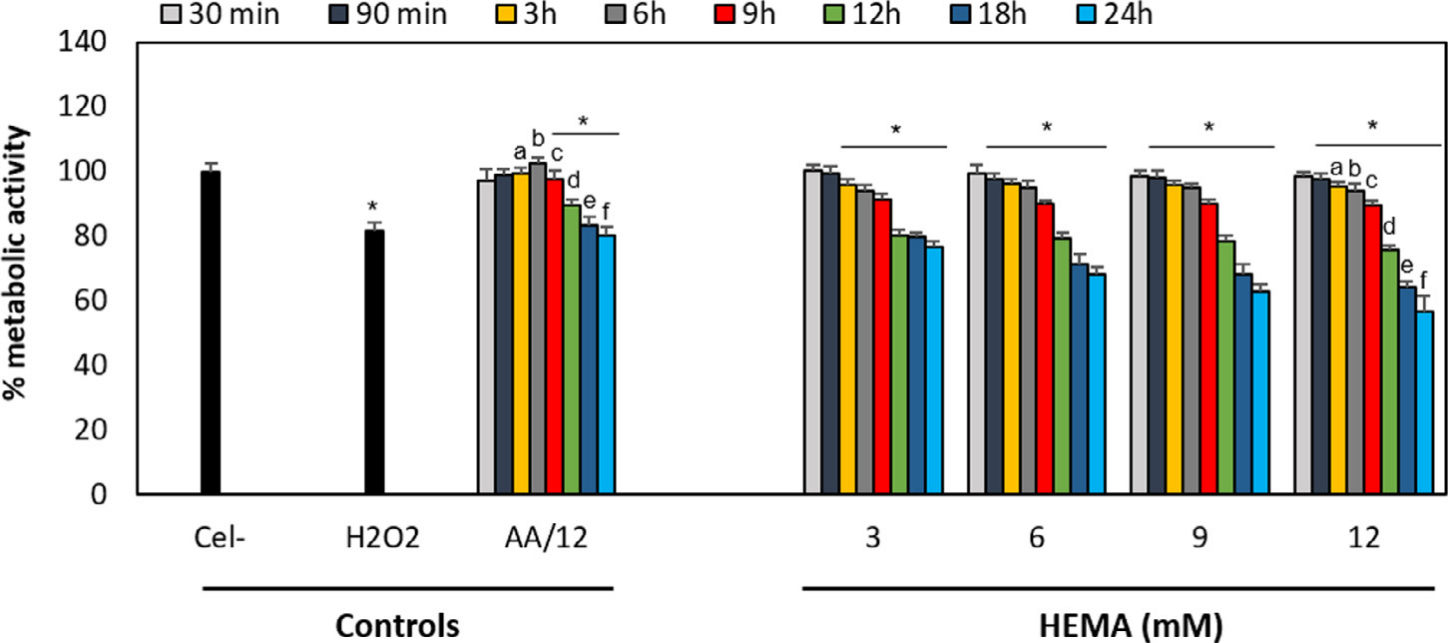
Effect of HEMA on hOLCs metabolic activity, measured by the resazurin assay. Cells exposed to 3, 6, 9, and 12 HEMA for 30 and 90 min, 3, 6, 9,  h. (C) Cells exposed to 3, 6, 9, and 12, 12, 18, and 24 h. After exposure, mitochondrial metabolic activity was detected by the resazurin assay and was quantified by spectrofluorometry at a wavelength of 535 nm_exc_/595 nm_ems_ in a TECAN reader. Data are shown as the mean ± SD of three independent experiments done in triplicate (*n* = 9). Each treatment group was compared to the 100% metabolic activity of the negative control (untreated cells), the metabolic activity percentage of the positive control (100 µM H_2_O_2_ for 4 h), and of the cells exposed to the highest HEMA concentration (12 mM) with 50 μg/mL of ascorbic acid (AA/12) for each time point. Asterisks indicate significant differences compared to the negative control (*p* < 0.05). Lowercase letters (a - f) indicate significant differences between treatment groups (*p* < 0.05).

**Fig. 6:**
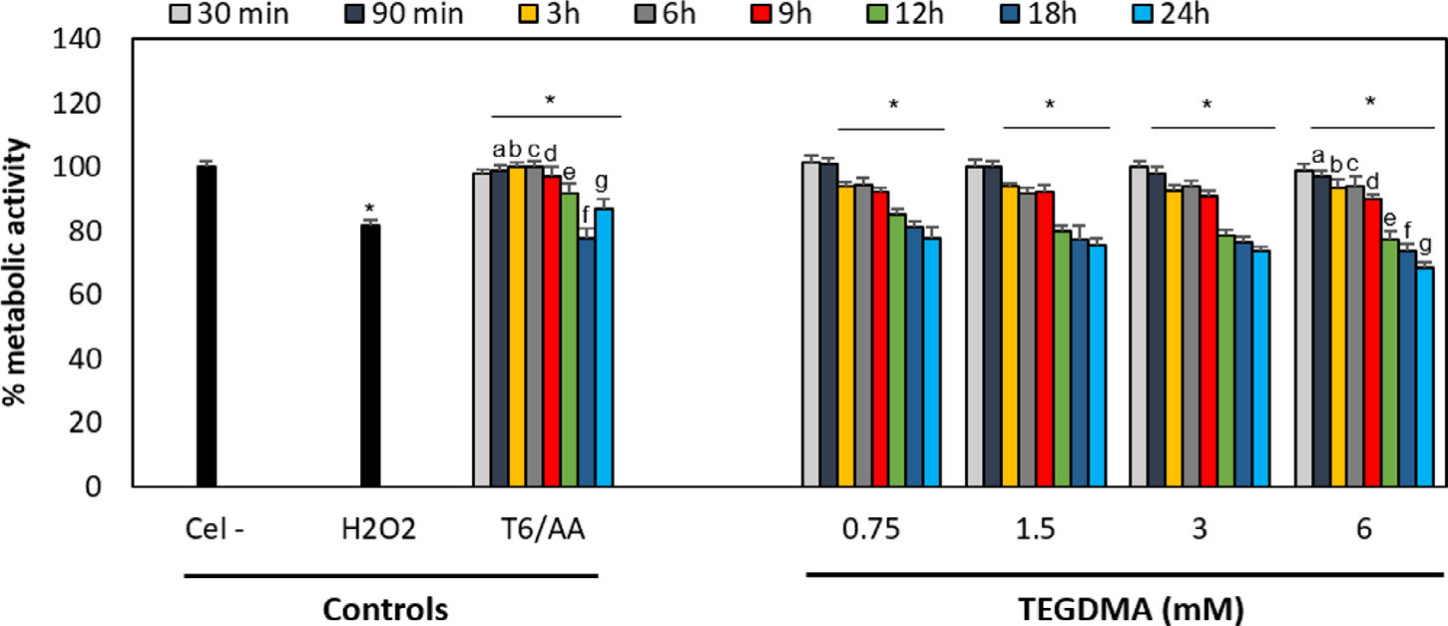
Effect of TEGDMA on hOLCs metabolic activity, measured by the resazurin assay. Cells exposed to 0.75, 1.5, 3, and 6 TEGDMA for 30 and 90 min. (B) Cells exposed to 0.75, 1.5, 3, and 6, 3, 6, 9,  h. (C) Cells exposed to 0.75, 1.5, 3, and 6 12, 18, and 24 h. Data are shown as the mean ± SD of three independent experiments done in triplicate (*n* = 9). Each treatment group was compared to the 100% metabolic activity of the negative control (untreated cells (Cel-)), the metabolic activity percentage of the positive control (100 µM H_2_O_2_ for 4 h), and of the cells exposed to the highest TEGDMA concentration (6) with 50 μg/mL of ascorbic acid (AA/6) for each time point. Asterisks indicate significant differences compared to the negative control (*p* < 0.05). Lowercase letters (a - g) indicate significant differences between treatment groups (*p* < 0.05).

Cells were incubated overnight at 37 °C in a 5% CO_2_ atmosphere in a 96-well flat-bottomed plate containing 25 × 10^3^ cells/well. The hOLCs were exposed to monomers and the medium was subsequently removed. One hundred microliters of 4.4 µM resazurin solution (R7017, Sigma-Aldrich), was added to each well. The plates were incubated for 3 h in a humidified atmosphere containing 5% CO_2_ at 37 °C. The plates were then read at a wavelength of 530 nm_exc_/590 nm_ems_ in a plate reader (Infinite M200, Tecan). The percentage reduction of resazurin to resorufin of the treated groups compared to the untreated control (100 %) was calculated for each exposure time. Three independent experiments were analyzed, each with three replicates (*n* =9) (Figs 4–6).

## Ethics Statement

Project approved by the ethics committee of the Facultad de Odontología, Universidad Nacional de Colombia (CIE-233-14).

## CRediT Author Statement

**Paula Baldion:** Conceptualization, Methodology, Resources, Investigation, Writing - Original Draft. **Myriam Velandia-Romero:** Conceptualization, Supervision, Writing - Review & Editing. **Jaime Castellanos:** Conceptualization, Supervision, Formal analysis, Writing - Review & Editing.

## Declaration of Competing Interest

The authors declare that they have no known competing financial interests or personal relationships which have or could be perceived to have influenced the work reported in this article.

## Data Availability

Data of the effect of dental resin monomers on human odontoblast-like cells (Original data) (Mendeley Data).
